# Molecular Dynamic Studies of Dye–Dye and Dye–DNA Interactions Governing Excitonic Coupling in Squaraine Aggregates Templated by DNA Holliday Junctions

**DOI:** 10.3390/ijms24044059

**Published:** 2023-02-17

**Authors:** German Barcenas, Austin Biaggne, Olga A. Mass, William B. Knowlton, Bernard Yurke, Lan Li

**Affiliations:** 1 Micron School of Materials Science and Engineering, Boise State University, Boise, ID 83725, USA; 2 Department of Electrical and Computer Engineering, Boise State University, Boise, ID 83725, USA; 3 Center for Advanced Energy Studies, Idaho Falls, ID 83401, USA

**Keywords:** DNA nanotechnology, molecular dynamics, dye aggregates, excitonic coupling

## Abstract

Dye molecules, arranged in an aggregate, can display excitonic delocalization. The use of DNA scaffolding to control aggregate configurations and delocalization is of research interest. Here, we applied Molecular Dynamics (MD) to gain an insight on how dye–DNA interactions affect excitonic coupling between two squaraine (SQ) dyes covalently attached to a DNA Holliday junction (HJ). We studied two types of dimer configurations, i.e., adjacent and transverse, which differed in points of dye covalent attachments to DNA. Three structurally different SQ dyes with similar hydrophobicity were chosen to investigate the sensitivity of excitonic coupling to dye placement. Each dimer configuration was initialized in parallel and antiparallel arrangements in the DNA HJ. The MD results, validated by experimental measurements, suggested that the adjacent dimer promotes stronger excitonic coupling and less dye–DNA interaction than the transverse dimer. Additionally, we found that SQ dyes with specific functional groups (i.e., substituents) facilitate a closer degree of aggregate packing via hydrophobic effects, leading to a stronger excitonic coupling. This work advances a fundamental understanding of the impacts of dye–DNA interactions on aggregate orientation and excitonic coupling.

## 1. Introduction

Dye aggregates can exhibit exciton delocalization, or the quantum mechanical sharing of a local excited state among dyes. This behavior has applications ranging from photovoltaics [1], imaging [2], and nanoscale computing [3,4]. The quantum nature of the coherent delocalized exciton transfer can be exploited in the implementation of quantum gates [5]. Controlling exciton delocalization, then, is analogous to controlling the qubit. However, creating systems capable of reliable dye-dye exciton delocalization involves manipulating dye-dye interactions to promote controlled dye aggregation. The search for organic materials with tailored electronic and optical properties has given rise to interest in the methods that can be used to control exciton delocalization between dyes. These organic electronic materials take advantage of the fact that dyes can be optically excited and that the excited state can share the generated exciton to other nearby dyes [6,7,8,9].

**Figure 1:**
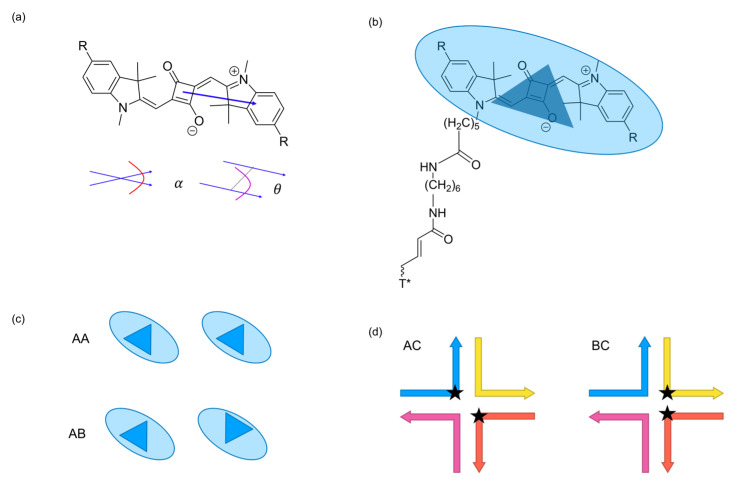
Molecular design of squaraine (SQ) dyes covalently attached to a DNA Holliday junction (HJ). (**a**) A SQ dye with an arrow representing $\hat{\mu }$ as it points from the center of the dye to the center of benzene ring. The SQ dyes in this study are SQ-H2 (R = H), SQ-Cl2 (R = Cl), and SQ-Me2 (R  = CH3). The oblique angle α and slip angle θ between the transition dipole vectors of two dyes are also shown. (**b**) The SQ dyes, attached to DNA via a linker. The SQ dye attaches to the linker, and the linker requires an extra T base to attach to the DNA. The SQ dye is represented as a blue oval with a dark blue triangle to indicate the orientation of the packing arrangement. (**c**) Schematic indication of dye arrangement, where AA refers to the parallel dye arrangement (i.e., face to face) while AB refers to antiparallel dye arrangement (i.e., face to back). (**d**) Transverse AC and adjacent BC dimer HJ configurations. The locations of the dyes are marked with a star. Each arrow represents a DNA strand of the HJ, where the top left blue arrow is strand A, the top right yellow arrow is strand B, the bottom right orange arrow is strand C, and the bottom left pink arrow is strand D. All arrows are pointing from the 5′ to 3′ direction.

The foundational theory of exciton delocalization in dye aggregates stems from the work of Frenkel, Davydov, and Kasha, with insight into why dyes couple, how dye orientations impact aggregate properties, and how the behavior of the exciton is controlled from these inputs [10,11,12,13]. The exciton hopping parameter, $J_{m,n}$, was introduced to quantify the strength of dye coupling from what is known as the Kasha molecular exciton model [14]. A practical approximation of $J_{m,n}$, known as the dipole approximation, simplifies the excitonic coupling between a pair of dyes as a dipole–dipole interaction energy between the dye transition dipole moments, μ. The interaction energy depends on their constituent dye orientations and relative positions [15,16]. In addition, dye orientations can be determined by the position of μ relative to the other dye in the aggregate. Aggregates can be categorized via the light absorption behavior relative to the dye monomer. These categories include: the J-aggregate configuration, in which the μ vectors for two dyes are aligned head-to-tail, and the dimer absorption spectrum red-shifts relative to the dye monomer; the H-aggregate configuration, in which the μ vectors for two dyes are aligned face-to-face, and the dimer absorption spectrum blue-shifts relative to the dye monomer; and a special classification known as the “oblique” dimer, in which the μ vectors of two dye monomers are perpendicular to each other. The oblique dimer has a unique absorption spectrum in which the absorption peak splits relative to the monomer, in a phenomenon known as Davydov splitting [17]. Indeed, dye orientation occupies an essential parameter in dye coupling. In the extended dipole approximation, the dye orientation is captured in Equation (1) [18].

(1) $$J_{m,n}=J_{o}\left(\frac{1}{\left|r_{1}-r_{2}\right|}-\frac{1}{\left|r_{1}-s_{2}\right|}-\frac{1}{\left|s_{1}-r_{2}\right|}+\frac{1}{\left|s_{1}-s_{2}\right|}\right)$$

(2) $$J_{o}=\frac{{\left|\mu \right|}^{2}}{4\pi \epsilon_{0}n^{2}l_{m}l_{n}}$$

where |μ| is the magnitude of the transition dipole, $\epsilon_{0}$ is vacuum permittivity, n is the refractive index of the medium, and $l_{im}$ and $l_{n}\;$ are the lengths of π conjugation bridges of dyes m and n, respectively. The π conjugated bridge is defined as the unbroken chain of overlapping p-orbitals within a conjugated organic compound. The π conjugation bridge is primarily responsible for the light absorbing properties of dyes. In the extended dipole approximation, the ends of the π conjugation bridge are labeled as $r_{i}$ and $s_{i}$, where the i denotes individual dyes. Figure 1a shows the direction of the π conjugation bridge. In addition, the dimer packing arrangement should be considered. In this paper, two distinct packing arrangements that have been studied are referred to as parallel or antiparallel packing, or as AA or AB, respectively. These packing arrangements can also be considered as face to face, or face to back, and they are illustrated in Figure 1b,c.

DNA nanotechnology has recently demonstrated that it is a useful tool for the assembly of nanoscale structures. It can control dye-dye distances with sub-nanoscale control of distances necessary to produce excitonic coupling in dye aggregates covalently templated (or scaffolded) by DNA [19,20,21,22,23,24,25,26,27,28,29,30,31,32,33]. The construction of complex DNA scaffolds is facilitated by the programmable self-assembly method enabled by Watson–Crick–Franklin base pairing rules [34,35]. More complex DNA scaffolds can be used to create molecular aggregates [23,36,37,38]. The four-arm DNA Holliday Junction (HJ) is an example [37,38,39]. The arms of the DNA HJ are beneficial in preventing the dimerization of dsDNA labeled with hydrophobic dyes [40]. In addition, the HJ enables the creation of higher-order aggregates, such as trimers and tetramers, by covalently attaching the dyes to the core of the HJ [41]. The structure of the HJ has been well characterized, giving insight into how dye aggregates might interact with the scaffold [42,43]. However, the atomistic-scale characterization of HJ modifications from additional ligands is lacking and would be extremely useful because the HJ can be considered as one of the basic building blocks for a DNA–nanobreadboard excitonic device [40]. The HJ also promotes a greater design space to study the J (i.e., head-to-tail alignment) and H (i.e., face-to-face alignment) dye aggregates over a DNA duplex.

In DNA-templated dye aggregates, non-covalent dye-dye interactions govern the excitonic coupling and aggregate geometry [44]. Our group has studied these interactions in prior experiments by explicitly testing the effects of varying hydrophobic substituents on the squaraine (SQ) dye [45]. The SQ dye is of great research interest due to their customizability and enhanced photophysical properties from substituents [46,47,48]. Substituents can also impact μ by augmenting their electronic donor or withdrawing behaviors, which can be quantified by the Hammett constant, $\sigma_{p}\;$ [49,50]. Additionally, dye aggregates in HJs have been further modified by the placement of supramolecular rotaxane rings around SQ dyes that promote the elusive oblique dimer of hydrophilic SQ dyes [51,52,53]. We have, thus far, explored how dye-dye interactions can be tuned to promote more favorable dye packing in aggregates attached to HJ. An additional consideration of the supramolecular interactions between a dye and DNA; in particular, the specifics of dyes binding to DNA influenced by dye sterics and hydrophobicity, will facilitate the rational design of dyes for molecular aggregates templated by DNA.

Computational modeling can provide detailed insight into the dye–DNA systems discussed above. Our group has successfully used the Kühn–Renger–May (KRM) modeling of the experimental optical spectrum to extract the dye aggregate structures templated by DNA scaffolds, and thereby to extract the degree of excitonic coupling between pairs of dyes within the aggregates [14,45]. KRM modeling has provided valuable insight into dimer orientations, but it requires experimental optical data as an input. Density Functional Theory (DFT) has already been employed to study excitonic coupling in dyes. It has also been used to computationally screen for promising dye candidates, as presented in our previous DFT survey of dyes [49,50,54]. Expanding the simulations to accommodate DNA, specifically a HJ, is computationally expensive for DFT, so an alternative simulation technique is required.

Molecular Dynamics (MD) is an alternative, less computationally costly method to study dye aggregate–DNA configurations. Initial efforts to use MD to model dye–DNA systems, containing the commercially available and extensively characterized cyanine (Cy) dyes, have been conducted. Stennet et al. studied a Cy3 monomer bonded to a DNA duplex backbone. They found that MD produced expected Cy3-DNA conformers when compared to their time-resolved fluorescence experiments [55]. Cunningham et al. studied a Cy3 dimer on a DNA duplex. They used MD to calculate the orientation factor, κ, in order to predict dimer stacking configurations and Cy3-DNA interactions [56]. Our prior MD simulations predicted Cy5 and Cy5.5 dimer orientations on a DNA HJ [57]. We found that both Cy5 and Cy5.5 were the bigger dyes than Cy3, but these dyes were still able to intercalate into a DNA HJ. In these cases, interaction was seen as favorable because it encouraged closer dye distances, and MD proved a valuable method to study this phenomenon. Our prior studies also demonstrated that dye orientations and associated exciton hopping parameters, $J_{m,n}$, derived from the MD trajectories (Equation (1)), agreed well with experimental measurements. Therefore, MD simulations can provide insight into behavior that is difficult to measure experimentally.

Our prior work successfully demonstrated that MD could predict Cy dimer orientations and their interactions with DNA HJ [58]. Studies, however, are lacking for the MD modeling of SQ dye aggregates on a DNA HJ template. Additionally, prior MD simulations of Cy dyes on DNA provide us with the methods to characterize these dye–DNA interactions, and a potentially new source of heterogeneity from dye parallel or antiparallel arrangement. Here, we continued using MD to study SQ dye–DNA HJ systems. Cl- and Me- substituents were also chosen to investigate how sensitive the excitonic coupling was to even very small changes in dye hydrophobicity. These SQ variants were chosen to promote a greater hydrophobicity of the SQ dye, thus improving the dimer packing distances, and potentially achieving the *J_m__,n_* values of SQ that are comparable to and even larger than those of the Cy dimers [14]. Here, we present the results of using MD to study SQ-H_2_, SQ-Cl_2_, and SQ-Me_2_ transverse (i.e., AC) and adjacent (i.e., BC) dimers, as shown in Figure 1d. There are 6 dimers in total. Each dimer has two packing arrangements for 12 different configurations in total. MD was used to predict SQ dimer orientations relative to the DNA HJs using time series average and clustering methods for comparison. Using the transition dipole moments calculated with DFT in our prior work, and the orientations of the SQ dyes obtained from MD, we estimated the corresponding *J_m__,n_* value of each dimer [50]. The computational results were validated with the experimental data. This work could provide insight into SQ dimer packing behavior for adjacent and transverse dimers, parallel and antiparallel arrangements, and SQ dyes with different substituents. We found that the dimer configuration could affect excitonic coupling by analyzing dye–dye and dye–DNA interactions.

## 2. Results

### 2.1. Dimer Orientation

To classify dimer orientations, we estimated the dye-dye distance R, the oblique angle α, the slip angle θ, the orientation factor $\kappa^{2}$, and the packing orientation factor $\kappa^{\prime }$ where:

(3) $$\alpha =\cos^{-1}\left({\hat{\mu }}_{m}\cdot {\hat{\mu }}_{n}\right)$$

(4) $$\theta =\cos^{-1}\left(\hat{R}\cdot {\hat{\mu }}_{n}\right)$$

(5) $$\kappa^{2}={\left({\hat{\mu }}_{m}\cdot {\hat{\mu }}_{n}-3\left({\hat{\mu }}_{m}\cdot \hat{R}\right)\left({\hat{\mu }}_{n}\cdot \hat{R}\right)\right)}^{2}$$

(6) $$\kappa^{\prime }={\hat{\mu }}_{m}\cdot {\hat{\mu }}_{n}$$

To compare with the experimental data, the value of α above 90° is defined as: α=|α−90| if α>90°. Similarly, for θ, θ=|θ−90| if θ>90°.$\;{\hat{\mu }}_{i}$ are the unit vectors of transition dipole moments μ  of dyes m and n, and $\hat{R}$ is the unit vector of the dye–dye distance R from dye m to dye n. As $\kappa^{2}$ approaches 1, the dimer configuration approaches like an H-aggregate (i.e., face-to-face alignment). As $\kappa^{2}$ approaches 4, the dimer configuration approaches like a J-aggregate (i.e., head-to-tail alignment). $\kappa^{\prime }$ is used to verify whether the initialized packing orientation is maintained throughout the simulation, by taking advantage of the dipole always pointing along the same direction of the dye. Therefore, the dot product of $\hat{\mu }$ will be positive if the vectors are in a parallel packing, or negative if in an antiparallel packing.

The angles α and θ are illustrated in Figure 1a. The orientation and position values for the dyes are defined with the following atoms: The Center of Mass (CoM) of the dyes is defined as the CoM of the four carbon atoms of the squarate moiety. The distance between the CoMs of dyes is defined as R. The transition dipole unit vector, $\hat{\mu }$, is defined as a vector that starts at the CoM of a dye as its origin, and points towards the center of the benzene ring of the squarate moiety furthest from the linker. This direction is chosen to place $\hat{\mu }$, pointing along the long axis of the dye. $\hat{R}$ is the unit vector pointing from the center of dye m to n. In this study, we assume that the ends of the π conjugation bridge terminals are at the center of the benzene groups at the ends of the dye (Figure 1a). They are labeled as $r_{i}$ and $s_{i}$ where i denotes individual dyes. These definitions are used to match the values generated in Mass et al. [14]. The chemical structure of the SQ dye with a linker is given in Figure 1b.

Dimer type, whether a J- or H-aggregate, is an important distinction when predicting dye aggregates. Mass et al. observed an H-aggregate, where the transition dipole moments were stacked relative to another [14]. These results were generated using KRM modeling; however, it remains unknown how the dyes orient themselves relative to the DNA, and how the dimer–DNA configuration changes with time. The DNA–dye relationship is left ambiguous from KRM modeling alone, and so MD can study this interaction.

**Figure 2:**
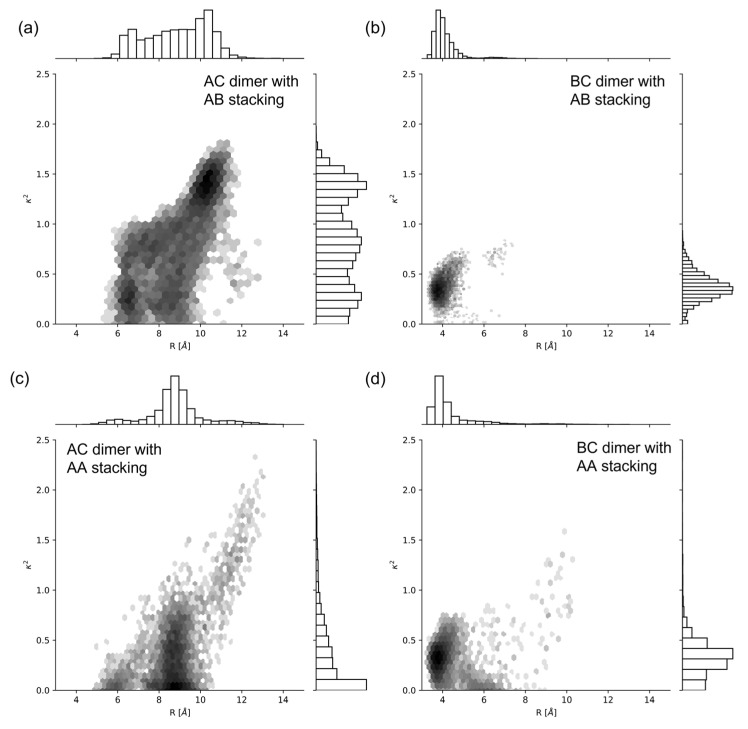
Two-dimensional histogram plots of R, compared to $\kappa^{2}$ for SQ-H2. (**a**) AC dimer and AB stacking, (**b**) BC dimer and AB packing, (**c**) AC dimer and AA packing, (**d**) BC dimer and AA stacking. The log color scale represents the population of bins of the histogram, where darker colors convey a more populated bin, and lighter colors convey a less populated bin. MD snapshots for the corresponding SQ-H2 configurations are shown in Figure 3.

We used SQ-H_2_ transverse (i.e., AC) and adjacent (i.e., BC) dimers attached to DNA HJs as a reference to compare to SQ dyes with other constituents (Figure 1d). We chose SQ-H_2_ as a benchmark to highlight the effects of packing behavior AA vs. AB (Figure 1c) and substitution, e.g., substituting H with Cl and Me, on dye–DNA interaction and excitonic coupling properties. The AC and BC dimers differ by the dye attachment positions on the HJ. Dye packing has been shown experimentally to be sensitive to the dye attachment positions within a HJ [14,41,45]. The AA and AB configurations represent parallel and antiparallel dye alignment in the dimers. First, we investigated orientation distribution of dyes for each dimer alignment based on orientation factor $\kappa^{2}$. Figure 2 shows the histograms of R and $\kappa^{2}$ for each SQ-H_2_ dimer alignment, where a darker color represents a greater density of values in a bin, and lighter colors represent less values in a bin. The distribution of values for each axis is also shown as bar charts corresponding to their axis. There is a more prominent normal distribution of values for the BC dimers compared to the AC dimers (as seen in Figure 2b,d vs. Figure 2a,c), representing a stronger correlation between R and $\kappa^{2}$ for the BC dimers than the AC dimers. When comparing the bar charts, parallel dye arrangement AA tends to have less distribution of orientation compared to antiparallel dye arrangement AB. These results suggest that the SQ dimer orientation is sensitive to the dye attachment to the HJ. The BC dimer in particular shows a stronger correlation between R and $\kappa^{2}$ than the AC dimer.

**Figure 3:**
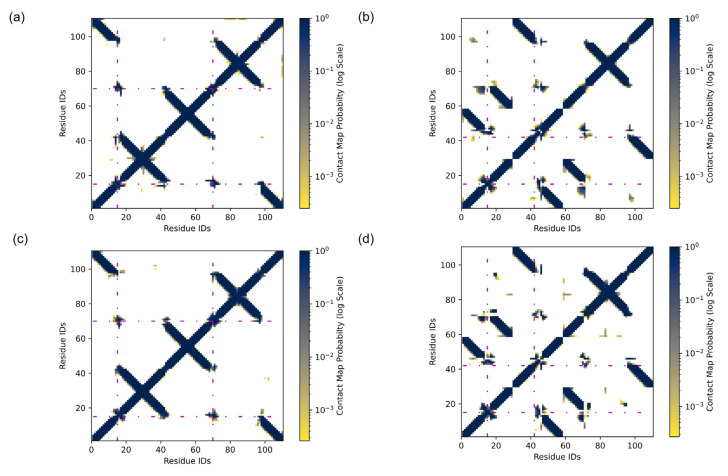
Contact maps for SQ-H_2_ (**a**) AC dimer and AB stacking, (**b**) BC dimer and AB stacking, (**c**) AC dimer and AA stacking, and (**d**) BC dimer and AA stacking. For the AC dimer, residue IDs 15 and 70 represent the dyes. For the BC dimers, residue IDs 15 and 42 represent the dyes. The dye residues are marked by magenta dashes. Contact map probability is represented by the log color scale, where darker colors represent more contact time, and lighter colors represent less contact. MD snapshots for corresponding SQ-H_2_ configurations are shown in Figure 4.

To further analyze why the AC dimer has the wider R distribution than the BC dimer, we generated contact maps for the dye–DNA system (Figure 3). Contact maps, which condense the complex three-dimensional dynamic molecular structure into more accessible two-dimensional figures, have been commonly used to explain protein dynamic structures to study protein conformational flexibility and structure [59,60,61]. As a pilot study, we applied contact maps to various SQ dye–DNA systems to advance our understanding on how the SQ-H_2_ dimer interacts with DNA and how the dimer packing behavior, i.e., AA vs. AB, and attachment points within the HJ, i.e., AC vs. BC, influence the interaction. Figure S1 shows unique IDs that identity distinct positions in the AC dimer–DNA HJ system and the BC dimer–DNA HJ system. Each ID number corresponds to a group of atoms that could make up a DNA base pair, a linker, or a dye, as a residue in the contact map. For example, in the AC dimer–DNA HJ system, the residue IDs for the A strand start with number 1 and end with number 28 while the B strand starts with number 29 and ends with number 55, as shown in Figure S1a. In the BC dimer–DNA HJ system, the residue IDs for the A strand start with number 85 and end with number 111 while the B strand starts with number 1 and ends with number 28, as shown in Figure S1b. We assigned the residue IDs based on the dye attachment positions on the DNA HJ. A circle is drawn around residue 15, which corresponds to the position for one of the dyes at the DNA junction. This circle represents a contact circle with a radius of 1.2 nm in the AC dimer–DNA HJ system or the BC dimer–DNA HJ system. During a MD simulation, if another residue appears within this circle, this residue would be considered in contact with residue 15. This idea is applied to all the residues to create the contact map for the entire dye–DNA system. Residue IDs are plotted along the x and y axes, as shown in Figure 3, and the x and y axes values are the same. The x and y axes therefore represent every pair of residues, and a coloring system is used to represent each pair’s contact probability, i.e., the probability of a residue appearing in a circle with a radius of 1.2 nm. Due to the dynamic nature of MD simulation, some residues may be in contact, i.e., appearing in a contact circle, throughout the whole simulation time, part of the time, or not at all. This contact probability is represented by the shading of each point, where the darker colors represent more contact throughout the simulation time, and the lighter colors represent less contact throughout the simulation time. No color means no contact within the simulation time. More information on the contact criteria and residue definitions can be found in the Materials and Methods section.

The DNA could influence the dimer orientation distribution. For example, the sustained hydrogen bonds that stabilize the double helix of the DNA strands are observed as dark diagonals in Figure 3. The diagonals are an expected result for a DNA contact map and reveal that the DNA maintains its helical structure within both the AC and BC HJ throughout the simulation. The dye residue IDs are emphasized with a magenta dotted line to allow for a focused examination of the dye–DNA contact. For the AC dimer, residue IDs 15 and 70 represent the dyes. For the BC dimers, residue IDs 15 and 42 represent the dyes. For example, the circles drawn at residue 15 in Figure S1a,b are represented as magenta dashed lines at residue ID 15 on the x and y axes in Figure 3a,b, respectively.

To better visualize and interpretate Figure 3a,b, we have a larger version for both figures as shown in Figures S2 and S3 with alphabetical labels on regions of interest, respectively. Residue 15 in the AC dimer–DNA HJ system has more residue pairs with contact than that in the BC dimer DNA HJ system because Figure S2 shows more dark color spots along the magenta dashed line labeled with A, B and C at residue 15 compared to Figure S3 with only one dark color spot labeled with A. Label A in Figure S2 corresponds to the strand D near the crossover region of the HJ in Figure S1a. Label B corresponds to the C strand near the crossover region including the other dye on the C strand. Label C corresponds to the B strand near the crossover region of the HJ. Figure S3 shows labels A and B. Label A suggests a contact of the dye on the B strand and the residues near the crossover region of the C strand, including the other dye in Figure S1b. Label B suggests the dye on the C strand contacting the residues near the crossover region of the D strand. Therefore, the dyes in the AC dimer have a greater probability of contact with the surrounding DNA residues compared to the BC dimers. This trend can be read qualitatively by observing that the number of residues that have contacts with the dyes along the magenta line of Figure 3, showing more contacts in AC (residue IDs 15 and 70 in Figure 3a,c) than those in BC (residue IDs 15 and 42 in Figure 3b,d).

The contact probability is shown as a color scale of each plot in Figure 3. Both AC and BC HJs show 100% contact probability between dye–dye residues. Considering dye–DNA contact, most of the residue contacts occur in both AC and BC HJs are towards the junction region. The AC dimer also shows that some residue contacts occur farther from the junction region than the BC dimers does, as evidence showing the higher probability of residues along the magenta dash lines.

**Figure 4:**
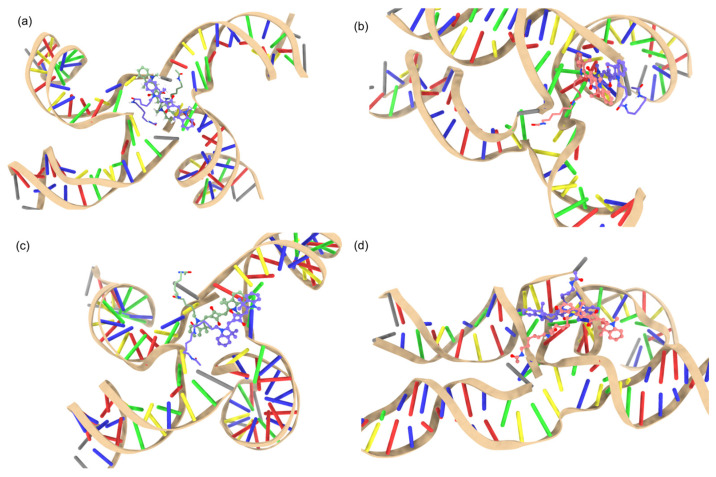
MD snapshots of SQ-H2. (**a**) AC dimer and AB stacking, (**b**) BC dimer and AB stacking, (**c**) AC dimer and AA stacking, (**d**) BC dimer and AA stacking.

Figure 4 shows more evidence for the dye–DNA contacts in the snapshots of the MD simulations by providing structural details. We found that the AC dimer tends to encourage a more open HJ (Figure 4a,c) as opposed to the closed HJ for the BC (Figure 4b,d) dimer. Figure 2 and Figure 4 show that the AC dimers have a wider distribution of orientations and distances because of their higher contact with DNA compared to the minimal DNA contact and smaller distribution for the BC dimers. The wider distribution of orientations and distances are noted by a wider range of values shown along the x and y axes of Figure 2, and the difference in HJ structure shown in Figure 4. Such difference can be attributed to the HJ being open or closed. The open HJ allows for more mobility of the AC dimer as opposed to the closed HJ that limits the BC dimer mobility. The wider distribution of orientations and distances also implies a possibility for a different configuration of the HJ with the AC dimer compared to the BC dimer. We also found that the BC dimers show a tendency to move away from the junction of the HJ while the AC dimers tend to stay within the center of the HJ, agreeing well with the contact map of the AC HJ showing more dye–DNA contacts than that in the BC HJ (Figure 3).

**Figure 5:**
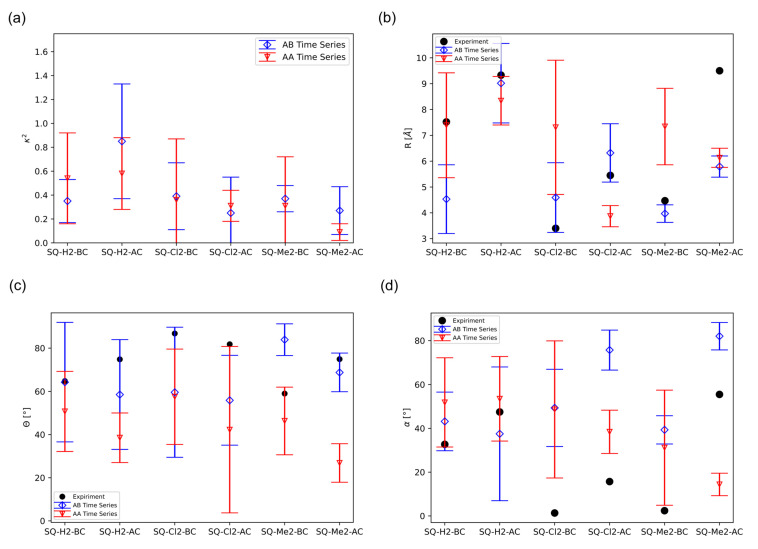
Orientation values for each dimer and packing from Time Series data, including (**a**) Orientation factor $\kappa^{2}$, (**b**) Dye–dye distance R, (**c**) Slip angle θ, and (**d**) Oblique angle α. The experimental values are adapted from Mass et al. [14].

To further investigate the effects of substituents and packing types of each dimer, we compared the $\kappa^{2}$ and R values for each SQ dimer obtained from our MD simulations, as shown in Figure 5a,b. The average computed $\kappa^{2}$ and R values for each dimer are represented as a mark, and their standard deviations are shown as lines extended from the average marker. The “Time Series” represents that the values are the average across the MD simulation time. More details can be found in the Materials and Methods section. The experimental values, reported by Mass et al., are also shown for comparison [14].

In general, the dyes fluctuate from being close to an H-aggregate to being oblique, regardless of AA or AB packing, as shown in Figure 5a. This observation is supported by the $\kappa^{2}$ values fluctuating between 0 and 1. An additional clustering method known as GROMOS clustering was used to reveal potential multistate ensembles from the MD trajectory. GROMOS clustering attempts at grouping frames of the MD simulation according to similar atomic position, which generates microstates found within the simulation. GROMOS is further explained in the Materials and Methods section, and the GROMOS data are reported in Figure S4a. GROMOS clustering follows a similar trend found in Figure 5a, except for the AA GROMOS values of the SQ-H_2_ dimers, where GROMOS predicts that SQ-H_2_-AC is closer to an H-aggregate than SQ-H_2_-BC. $\kappa^{2}$ is closer to 1 than to 4, suggesting more of an H-aggregate (i.e., a stacked configuration of two dyes), in agreement with Mass et al. [14]. The smaller standard deviation of the BC dimer $\kappa^{2}$ compared to the AC dimer $\kappa^{2}$ also supports the observations, as discussed before in Figure 2 and Figure 3, in which the BC dimers display a smaller distribution of orientations. The presence of the substituents promotes more perpendicular packing orientations than a pure H-aggregate, but the initial packing configuration is not preserved for some simulations, namely SQ-H_2_-BC AA and SQ-Cl_2_-BC AA. Figure S5 shows the packing orientation for the $\kappa^{\prime }$ average values and standard deviations of each HJ. The negative $\kappa^{\prime }$ values indicate that all HJs remain in AB packing as their original orientation. The HJ that starts with AA packing should have a positive $\kappa^{\prime }$ value, but SQ-H_2_-BC AA and SQ-Cl_2_-BC AA are predominately negative throughout the simulation. It should be noted that the BC HJs show less dye–DNA interactions (Figure 3 and Figure 4), so the flipping of the AA packing to the AB packing for these BC HJs suggests some propensity towards AB packing if there are minimal interactions between the dyes and their surroundings. Meanwhile, the AC HJs with AA packing maintain their initial packing conditions, so the DNA HJ may preserve packing if the dye–DNA interactions are allowed.

Next, we compared the R value for each dimer to the experimental data in Figure 5b. The general agreement with experimental trends indicates that the MD simulation approaches equilibrium conditions by averaging the time series alone. Further attempts to separate microstates from the GROMOS technique do not substantially improve the prediction, as shown in Figure S4b. The GROMOS results indicate that the GROMOS method, which in basic terms, is a filtering of states based on the Root Mean Square Deviation (RMSD), does not provide a clearer trend to the SQ dye ensemble analysis that we conducted in this study. With respect to the AA vs. AB packing, the general trend, as shown in Figure 5b, is that the AA dimers tend to have a larger R, except for SQ-Cl_2_-AC. This trend is expected because AA packing promotes steric clashing with the dimethyl groups on the indolenine moiety of the SQ dye. SQ-Me_2_-AC also deviates from experiments, with R from the simulation being much smaller.

The R and $\kappa^{2}$ values cannot fully address different degrees of freedom for dye orientation within the HJ. To further compare dimer orientations, θ and α angles were also calculated in comparison with the experimental data, as shown in Figure 5c,d, respectively. For the θ values of the AB packing, the Time Series data generally follow the trend of experimental data, as does the GROMOS data other than SQ-H_2_-AC and SQ-Cl_2_-AC (Figure S4c). The AB Time Series data produces a more similar trend to the experimental results than the AA Time Series data. AB GROMOS data generally follow the AB Time Series trend, except for the SQ-H_2_-AC and SQ-Cl_2_-AC data. The AA Time Series and the GROMOS data likewise follow similar trends. Overall, the θ value is within 50% of the experimental data. For the α values, MD predicted SQ-Me2 best, where the AB packing data are more in agreement with experimental data than the AA packing data. There was no significant difference between the trend presented in the Time Series trend of α shown in Figure 5d and the GROMOS α trend shown in Figure S4d. The reason for why there is a difference between the α values obtained from MD and from the experiments could be that the potential for steric clashing for planar dyes (α=0) was underrepresented in the phase space sampled using the MD method. Both θ and α values suggest that these dyes approach an H-dimer, but MD simulations predict a more perpendicular orientation, where the dyes lie in more of an “X” packing arrangement than a true H-dimer. Even so, the dyes are stacked on the standard deviation of angles support that the dimers are at least fluctuating in an H-dimer regime. 

Generally speaking, Figure 5 suggests that the dimer in a BC configuration tends to have a smaller R than that in an AC configuration. Similarly, substituents also promote a smaller dye distance compared to the unmodified SQ-H_2_ dimers. According to Mass et al. [14], the smaller dye distance could be the consequence of the hydrophobicity of the dye. In MD, hydrophobicity is not directly parametrized. Rather, electrostatic interactions are a manifestation of hydrophobic differences of dyes. The nonbonded parameters that govern these electrostatic interactions then adequately capture the dye coupling affinity of hydrophobic dyes.

### 2.2. Excitonic Coupling

**Figure 6:**
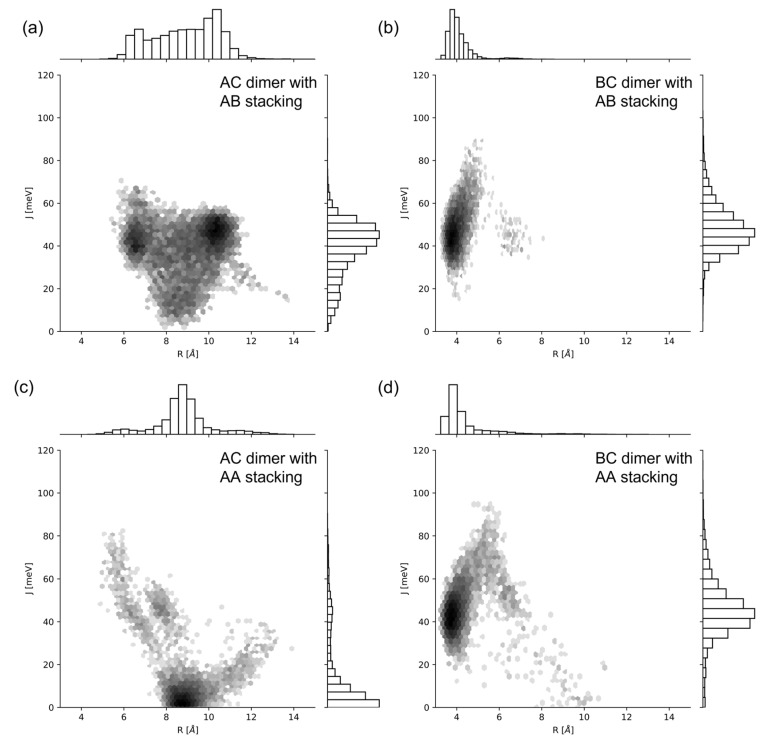
Two-dimensional histogram plots of R compared to $J_{m,n}$ for SQ-H2. (**a**) AC dimer and AB packing, (**b**) BC dimer and AB packing, (**c**) AC dimer and AA packing, (**d**) BC dimer and AA packing. The log color scale represents the population of bins of the histogram, where darker colors mean a more populated bin, and lighter colors means a less populated bin.

We calculated the exciton hopping parameter $J_{m,n}$ for each MD simulation trajectory using Equation (1). We sampled the Time Series data of $J_{m,n}$ as an ensemble average to represent the system. The additional GROMOS cluster method was performed on $J_{m,n}$ for comparison, where the average orientations from each cluster were used in Equation (1) to calculate a $J_{m,n}$ value associated with a cluster. The values for |μ| in Equation (2) were calculated using Time-Dependent Density Functional Theory (TDDFT) methods, given in Table 1. Figure 6 compares R vs. $J_{m,n}$ of SQ-H_2_ in a histogram, where darker bins represent more data in each bin, and lighter colors refer to less populated bins. The bar charts on the axis represent the one-dimensional distribution of each value. Like Figure 2, the AC dimers have a wider distribution of values compared to the BC dimer (as seen in Figure 6a,c vs. Figure 6b,d). The distribution of R and $J_{m,n}$ are not as wide in the AA compared to AB packing, but both distributions fall within the same bounds as shown on the bar charts of Figure 6. Packing therefore does not appear to contribute to the potential heterogeneity of a thermally accessible equilibrium ensemble, or if they did, they would not affect the final excitonic coupling of the BC dimers. The AC dimers appear sensitive to packing, where AB stacking promotes a higher $J_{m,n\;}$ than AA packing does.

**Figure 7:**
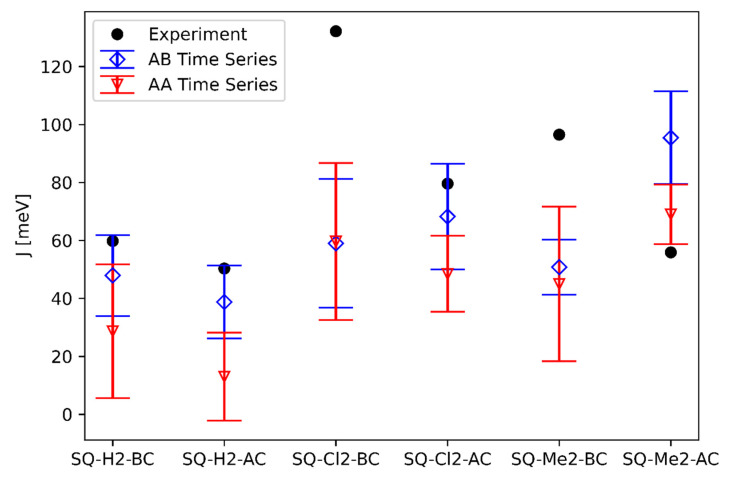
$J_{m,n}$ for all SQ aggregates Time Series average data with standard deviation bars for each simulation. The experimental values are adapted from Mass et al. [14].

As seen in Figure 7, the trend of the average $J_{m,n}$ for substituted SQ dyes in different packing generally follows trends from the experimental data. Additionally, the standard deviations of each analysis method are given as vertical lines that extend from the average value marked on the plot. Similar to R, the Time Series data more reliably match the trend and values from experimental data compared to the GROMOS results (shown in Figure S6). This also indicates that the simulation time average represents an ensemble average. Further processing with GROMOS does not substantially improve the comparison between experiment and simulation over that of the ensemble average. Substituent effects on $J_{m,n}$ align well with the R trend shown in Figure 5b. The smaller R values correspond to closer packing and greater $J_{m,n}$.

MD simulations suggest that hydrophobic dyes promote closer dimer packing, resulting in stronger excitonic coupling and thus larger $J_{m,n}$. In particular, SQ-Cl_2_ and SQ-Me_2_ have slightly larger hydrophobicity than SQ-H_2_ according to Mass et al. [14]. The hydrophobicity leading to larger $J_{m,n}$ prediction is supported by the experimental trend discussed by Mass et al. [14]. The trend for BC dimers to exhibit stronger coupling than the AC dimers is a result shown in Figure 3 and Figure 4, in which BC dimers are able to pack more closely, correlating to fewer dye–DNA interactions. For AA vs. AB packing, AB is closer to the experimental $J_{m,n}$ value than the AA packing, except for the SQ-Me_2_-AC values. The dimer $J_{m,n}$ values are within the simulation standard deviation, except for SQ-Cl_2_-BC and SQ-Me_2_-BC. The GROMOS clustering method does not show an improvement over the Time Series Average and is shown in Figure S6.

## 3. Discussion

Our MD simulations demonstrated that SQ dimers templated by HJs result in different excitonic coupling, depending on substituents, the dye attachment positions, and packing. We found that there is more dye–DNA contact in the AC dimer than in the BC dimer, which can lead to more heterogeneity being represented by the distribution of R and $\kappa^{2}$. Figure 2 and Figure 3 suggest that more dye–DNA contact promotes a wider distribution of orientation factor values, and the contact probability among neighboring residues is also wider for the AC dimers than the BC dimers. Figure 4 also provides a qualitative snapshot of the MD trajectory showing how the dyes would orient themselves relative to the DNA. The AC dimers tend to diffuse into the center of the HJ, and the BC dimers diffuse away from the HJ. Both SQ dimers resemble an H-aggregate, which is shown in the $\kappa^{2}$ values of Figure 2a as well as the $\kappa^{2}$ average values in Figure 5a. The dimer distance R is closer in a BC configuration than in the AC, which is also shown in Figure 2b and Figure 5b. Our previous study of photocrosslinking dye-labeled thymines showed that the dye intermolecular distance in these dimers stems from the distance between thymines to which the dyes are attached in the AC and BC configurations [62]. Our results suggest that the substituents on the SQ dyes promote the closer dimer distances when compared to the unmodified SQ-H2. Figure 2 and Figure 6 both demonstrate a wider distribution of orientation and distances in the AC dimers promotes lower $J_{m,n}$ compared to the BC dimers. Our results also suggest that, for the single-linker SQ dimers, the BC dimers promote greater excitonic coupling than the AC dimers do. We find more hydrophobic dyes lead to closer dye packing, which also promotes greater excitonic coupling as shown in Figure 7.

Our results capture dye structure-property relationships that suggest the choice of dye substituents can lead to a larger $J_{m,n}$, and determine possible sources for heterogeneity from dye–DNA interactions. These findings can help to guide dye design for desired excitonic applications. In future studies, we will continue to evaluate more advanced MD techniques with various advanced sampling methods using MD. A polarizable forcefield employed in MD may improve electrostatic interactions to encourage more planar coupling [63,64,65]. More advanced MD techniques, such as replica exchange, or Markov State Modeling (MSM), could allow for more refined sampling of the DNA–dye phase space [66,67].

## 4. Materials and Methods

### 4.1. Molecular Dynamics

MD trajectory data were generated using the GROMACS software package (version 2020.3), and custom force field potentials were created using the AMBER-DYES potentials from Graen et al. [68,69,70]. Using these parameters expedited tuning the simulations parameter details, as the SQ dye is very similar to the Cy dyes characterized in AMBER-DYES. Bonded parameters for the squarate moiety were repurposed from the AMBER-DYES, with appropriate atom types. The linker forcefield parameters were generated from the GAFF values [71]. The Restrained Electrostatic Potential (RESP) was used to generate the non-bonded values of the dyes and linkers [72]. In addition, the RESP method is sensitive to molecular conformer transitions, and previous work on the SQ dye showed unlikely conformer isomerization [50]. This makes the RESP method straightforward for generating non-bonded parameters for the SQ dye. The protocol used for RESP was described by the AMBER tutorials for setting up a DNA–ligand system [73].

Creating the initial configuration, often called the topology, presented an opportunity to test different SQ dimer orientations as a source of heterogeneity of dye-dye interaction effects on $J_{m,n}$. Specifically, while the experimental data produced an H-aggregate configuration, we did not know specifically how the packing occurred, which could impact the dimer orientations and be a source of heterogeneity. In this study, we refer to two packing, which we call AA or AB packing, as shown in Figure 1c. In the extended dipole approximation, we assume that the dye can be modeled as a 3D vector in space, but the MD trajectory must reconcile with SQ dye shape. When a dimer is stacked and the dyes appear parallel, we call it AA. When a dimer is stacked but the dyes appear antiparallel, they are called AB. Both AA and AB packing were created as the starting topology in the simulations. 

The following procedure was used to generate the starting topology for GROMACS. To begin with, the DNA was initialized using the Nanoengineer-1 software package (version 1.1.1) with no dye or linker [74]. This structure was then optimized using the in-package energy minimization with the Universal Force Field (UFF) [75]. Without the dye or linker, the resulting topology resembled the open HJ conformer. Then, the linker and dyes were added to the topology. Next, the HJ was made into the stacked HJ conformer. This was performed because the HJ is expected to prefer the stacked conformer, in which the junction collapses, in the experimental solvent environment. The criteria for making the stacked conformer came from previous MD HJ work that defined the Inter-duplex Angle (IDA) and the Torsion Angle (J) of a DNA HJ [42,43,76,77,78]. In addition to these criteria, another crucial feature of the stacked HJ is the extra co-facial packing in the DNA HJ junction. To satisfy the expected co-facial packing, the base pairs in the HJ junction were manually manipulated to stack. Finally, the stacked HJ were ready to be simulated via production MD. The dyes were placed on strand A and C to create a transverse dimer, and on B and C to create an adjacent dimer. Figure 1d illustrates the HJ and dye placements.

All MD simulations were performed using the stochastic dynamics integrator with a $\tau_{p}$ of 1 ps and a time step of 0.005 fs. Outputs were written every 5000 steps. AMBER14SB was used to describe the rest of the DNA and solvent surroundings in the NPT ensemble at 300 K in 15 mM MgCl_2_ to approximate experimental conditions [79]. The TIP3P water solvent model was used, and water molecules were removed when adding the salt counter ions. To prepare the NPT ensemble, the NVT ensemble was constructed, but all atoms in the SQ HJ were gradually equilibrated by reducing their position restraints from $1000\frac{\mathrm{kJ}}{\mathrm{mol}*{\mathrm{nm}}^{2}}$ to $500\;\frac{\mathrm{kJ}}{\mathrm{mol}*{\mathrm{nm}}^{2}}$, to no restraints. Electrostatic interactions were described using the Particle Mesh Ewald (PME) interaction, with a cutoff range of 12 Å. We also set the Van der Waal cutoff to 12 Å. Hydrogen bond constraints were applied using the LINCS algorithm [80]. The MD trajectory was generated using a stochastic dynamics integrator [81]. The trajectory was run for 2 μs to allow for equilbirum conditions. This time was chosen because DNA breathing is known to occur on the microsecond timescale, and this time has successfully simulated DNA movement [82].

MD can produce the orientations and positions necessary for the extend dipole approximation in Equation (1). The remaining term μ, needed to generate the exciton hopping parameter $J_{m,n}$, is an output of DFT and TDDFT calculations. Our previous DFT survey of SQ dyes also generated these μ values for SQ-H_2_, SQ-Cl_2_, and SQ-Me_2_ [50]. This study used μ generated in an implicit water solvent environment to mimic conditions that occur experimentally.

### 4.2. Data Analysis

After 2 μs, the output of the MD trajectory with no solvent was provided by GROMACS. The trajectory files were then read using the MDAnalysis python module [83,84]. All-atom coordinates were generated for every 50 timesteps, and the first 0.1 μs was skipped to account for a potential equilibration period as the system settled. The remaining time series data were analyzed for a variety of positions and orientation values over the time frame. In addition to using the time series data analyzed using MDAnalysis, the GROMOS clustering method contained within GROMACS was used to separate any noise or distinct states from the time series data [85]. GROMOS clustering works in principle by grouping the frames of the trajectory within a specified Root Mean Square Deviation (RMSD) of the atomic position cutoff. GROMOS clustering in this study was assigned a 0.05 nm cutoff to all atoms corresponding to both dye residues. Only the clusters that contained more than 5% of the total cluster amount were used to generate the GROMOS values. This cutoff was to discard the clusters that would be considered as transition states. For both methods, we reported the average value and the standard deviation of the simulation. For the Time Series datapoints, these values were generated by finding the average value over the entire 2 μs trajectory, as well as its standard deviation. The GROMOS average and standard deviation represent the average value of the generated GROMOS clusters, and the standard deviation of these cluster values.

Inspired by protein dynamics studies, we generated a contact map to investigate dye–DNA interactions [59,60,61]. For this study, we consider each DNA nucleotide as an individual component known as a residue, with a distinct identification number or residue ID. The additional T nucleotide, linker, and dye also have an individual residue ID. Residues are considered to be in contact if the center of mass of the residues are within 1.2 nm of each other. We chose 1.2 nm in this study because the electrostatic cutoff range is 1.2 nm, so there would be no nonbonded interactions beyond this distance in MD. This contact is counted for each residue between every other residue across the entire MD trajectory. The contact map probability counts the number of frames where a residue is in contact with another residue so that a residue within 1.2 nm for the entire simulation is 100% in contact with the other residue. The residue IDs of the AC and BC dimers differ, but their structures are the same. This means that the diagonal that represents the helical contact between DNA strands will not be the same between AC and BC due to different residue numbering. 

## 5. Conclusions

We used MD with a time series average and GROMOS clustering methods to investigate SQ transverse AC and adjacent BC dimer aggregates, and a variety of packing arrangements. We sought to determine whether the excitonic coupling of SQ dyes could be characterized with MD, and whether MD could provide a direct insight into the potential sources of heterogeneity observed in the experiments. We found that the SQ BC dimers interact less with the DNA HJ than the AC dimers do, resulting in a narrower range of orientations for the SQ dimers. However, all the SQ dimers are stable in an H-aggregate configuration. Additionally, dyes with hydrophobic substituents are coupled at smaller distances than those without hydrophobic substituents, promoting a greater excitonic coupling (i.e., a larger value for $J_{m,n}$). In comparison, between the time series average and the GROMOS clustering methods, the time series adequately represents the ensemble average of the DNA HJ. Our work will advance the development of dye–DNA systems for excitonic delocalization and associated applications, including DNA nanobreadboard devices.

## Figures and Tables

**Table 1:** |μ| values in unit of Debye (D) generated using Time-Dependent Density Functional Theory (TDDFT) adapted from Barcenas et al. [50].

Dye	|*µ*| [D]
SQ-H_2_	12.99
SQ-Cl_2_	13.41
SQ-Me_2_	13.73

## Data Availability

Select solvation energy and dipole moment data presented in this study is available in the Supporting Information. All other data presented are available upon request from the corresponding author. We also plan to make all data available in a publicly accessible data repository in the near future.

## Supplementary Materials

The following supporting information can be downloaded at: https://www.mdpi.com/article/10.3390/ijms24044059/s1.

## References

[1] Zheng C., Penmetcha A.R., Cona B., Spencer S.D., Zhu B., Heaphy P., Cody J.A., Collison C.J. (2015). Contribution of Aggregate States and Energetic Disorder to a Squaraine System Targeted for Organic Photovoltaic Devices. Langmuir.

[2] Umezawa K., Citterio D., Suzuki K. (2014). New Trends in Near-Infrared Fluorophores for Bioimaging. Anal. Sci..

[3] Sawaya N.P.D.D., Rappoport D., Tabor D.P., Aspuru-Guzik A. (2018). Excitonics: A Set of Gates for Molecular Exciton Processing and Signaling. ACS Nano.

[4] Wasielewski M.R., Forbes M.D.E., Frank N.L., Kowalski K., Scholes G.D., Yuen-Zhou J., Baldo M.A., Freedman D.E., Goldsmith R.H., Goodson T. (2020). Exploiting Chemistry and Molecular Systems for Quantum Information Science. Nat. Rev. Chem..

[5] Castellanos M.A., Dodin A., Willard A.P. (2020). On the Design of Molecular Excitonic Circuits for Quantum Computing: The Universal Quantum Gates. Phys. Chem. Chem. Phys..

[6] Runeson J.E., Lawrence J.E., Mannouch J.R., Richardson J.O. (2022). Explaining the Efficiency of Photosynthesis: Quantum Uncertainty or Classical Vibrations?. J. Phys. Chem. Lett..

[7] Engel G.S., Calhoun T.R., Read E.L., Ahn T.K., Mančal T., Cheng Y.C., Blankenship R.E., Fleming G.R. (2007). Evidence for Wavelike Energy Transfer through Quantum Coherence in Photosynthetic Systems. Nature.

[8] Rafiq S., Scholes G.D. (2018). From Fundamental Theories to Quantum Coherences in Electron Transfer. J. Am. Chem. Soc..

[9] Brookes J.C. (2017). Quantum Effects in Biology: Golden Rule in Enzymes, Olfaction, Photosynthesis and Magnetodetection. Proc. R. Soc. A Math. Phys. Eng. Sci..

[10] Frenkel J. (1931). On the Transformation of Light into Heat in Solids. Phys. Rev..

[11] Davydov A.S. (1948). Theory of Absorption Spectra of Molecular Crystals. Zh. Eksp. Teor. Fiz..

[12] Kasha M. (1991). Energy Transfer, Charge Transfer, and Proton Transfer in Molecular Composite Systems. Physical and Chemical Mechanisms in Molecular Radiation Biology.

[13] Kasha M. (1963). Energy Transfer Mechanisms and the Molecular Exciton Model for Molecular Aggregates. Radiat. Res..

[14] Mass O.A., Wilson C.K., Barcenas G., Terpetschnig E.A., Obukhova O.M., Kolosova O.S., Tatarets A.L., Li L., Yurke B., Knowlton W.B. (2022). Influence of Hydrophobicity on Excitonic Coupling in DNA-Templated Indolenine Squaraine Dye Aggregates. J. Phys. Chem. C.

[15] Abramavicius D., Palmieri B., Mukamel S. (2009). Extracting Single and Two-Exciton Couplings in Photosynthetic Complexes by Coherent Two-Dimensional Electronic Spectra. Chem. Phys..

[16] Madjet M.E., Abdurahman A., Renger T. (2006). Intermolecular Coulomb Couplings from Ab Initio Electrostatic Potentials: Application to Optical Transitions of Strongly Coupled Pigments in Photosynthetic Antennae and Reaction Centers. J. Phys. Chem. B.

[17] Davydov A.S.S.A.S. (1964). The Theory of Molecular Excitons. Uspekhi Fiz. Nauk..

[18] Czikklely V., Forsterling H.D.D., Kuhn H. (1970). Extended Dipole Model for Aggregates of Dye Molecules. Chem. Phys. Lett..

[19] Kuzyk A., Jungmann R., Acuna G.P., Liu N. (2018). DNA Origami Route for Nanophotonics. ACS Photonics.

[20] Dutta P.K., Varghese R., Nangreave J., Lin S., Yan H., Liu Y. (2011). DNA-Directed Artificial Light-Harvesting Antenna. J. Am. Chem. Soc..

[21] Albinsson B., Hannestad J.K., Börjesson K. (2012). Functionalized DNA Nanostructures for Light Harvesting and Charge Separation. Coord. Chem. Rev..

[22] Kringle L., Sawaya N.P.D., Widom J., Adams C., Raymer M.G., Aspuru-Guzik A., Marcus A.H. (2018). Temperature-Dependent Conformations of Exciton-Coupled Cy3 Dimers in Double-Stranded DNA. J. Chem. Phys..

[23] Jaekel A., Lill P., Whitelam S., Saccà B. (2020). Insights into the Structure and Energy of DNA Nanoassemblies. Molecules.

[24] Mazuski R.J., Díaz S.A., Wood R.E., Lloyd L.T., Klein W.P., Mathur D., Melinger J.S., Engel G.S., Medintz I.L. (2020). Ultrafast Excitation Transfer in Cy5 DNA Photonic Wires Displays Dye Conjugation and Excitation Energy Dependency. J. Phys. Chem. Lett..

[25] Asanuma H., Shirasuka K., Takarada T., Kashida H., Komiyama M. (2003). DNA-Dye Conjugates for Controllable H* Aggregation. J. Am. Chem. Soc..

[26] Mathur D., Kim Y.C., Díaz S.A., Cunningham P.D., Rolczynski B.S., Ancona M.G., Medintz I.L., Melinger J.S. (2021). Can a DNA Origami Structure Constrain the Position and Orientation of an Attached Dye Molecule?. J. Phys. Chem. C.

[27] Nicoli F., Roos M.K., Hemmig E.A., Di Antonio M., de Vivie-Riedle R., Liedl T. (2016). Proximity-Induced H-Aggregation of Cyanine Dyes on DNA-Duplexes. J. Phys. Chem. A.

[28] Roy S.K., Mass O.A., Kellis D.L., Wilson C.K., Hall J.A., Yurke B., Knowlton W.B. (2021). Exciton Delocalization and Scaffold Stability in Bridged Nucleotide-Substituted, DNA Duplex-Templated Cyanine Aggregates. J. Phys. Chem. B.

[29] Ruedas-Rama M.J., Orte A., Martin-Domingo M.C., Castello F., Talavera E.M., Alvarez-Pez J.M. (2014). Interaction of YOYO-3 with Different DNA Templates to Form H-Aggregates. J. Phys. Chem. B.

[30] Buckhout-White S., Spillmann C.M., Algar W.R., Khachatrian A., Melinger J.S., Goldman E.R., Ancona M.G., Medintz I.L. (2014). Assembling Programmable FRET-Based Photonic Networks Using Designer DNA Scaffolds. Nat. Commun..

[31] Wang M., Silva G.L., Armitage B.A. (2000). DNA-Templated Formation of a Helical Cyanine Dye J-Aggregate. J. Am. Chem. Soc..

[32] Boulais É., Schlau-Cohen G.S., Woodbury N.W., Yan H., Aspuru-Guzik A., Bathe M., Sawaya N.P.D., Veneziano R., Andreoni A., Banal J.L. (2018). Programmed Coherent Coupling in a Synthetic DNA-Based Excitonic Circuit. Nat. Mater..

[33] Fujii T., Kashida H., Asanuma H. (2009). Analysis of Coherent Heteroclustering of Different Dyes by Use of Threoninol Nucleotides for Comparison with the Molecular Exciton Theory. Chem. A Eur. J..

[34] Rauch S., Dickinson B.C. (2017). Programmable RNA Binding Proteins for Imaging and Therapeutics. Biochemistry.

[35] de Alba Ortíz A.P., Vreede J., Ensing B. (2022). Sequence Dependence of Transient Hoogsteen Base Pairing in DNA. PLoS Comput. Biol..

[36] Hart S.M., Banal J.L., Castellanos M.A., Markova L., Vyborna Y., Gorman J., Häner R., Willard A.P., Bathe M., Schlau-Cohen G.S. (2022). Activating Charge-Transfer State Formation in Strongly-Coupled Dimers Using DNA Scaffolds. Chem. Sci..

[37] Adendorff M.R., Tang G.Q., Millar D.P., Bathe M., Bricker W.P. (2022). Computational Investigation of the Impact of Core Sequence on Immobile DNA Four-Way Junction Structure and Dynamics. Nucleic Acids Res..

[38] Seeman N.C. (1982). Nucleic Acid Junctions and Lattices. J. Theor. Biol..

[39] Kallenbach N.R., Ma R.-I., Seeman N.C. (1983). An Immobile Nucleic Acid Junction Constructed from Oligonucleotides. Nature.

[40] Cannon B.L., Kellis D.L., Davis P.H., Lee J., Kuang W., Hughes W.L., Graugnard E., Yurke B., Knowlton W.B. (2015). Excitonic AND Logic Gates on DNA Brick Nanobreadboards. ACS Photonics.

[41] Huff J.S., Turner D.B., Mass O.A., Patten L.K., Wilson C.K., Roy S.K., Barclay M.S., Yurke B., Knowlton W.B., Davis P.H. (2021). Excited-State Lifetimes of DNA-Templated Cyanine Dimer, Trimer, and Tetramer Aggregates: The Role of Exciton Delocalization, Dye Separation, and DNA Heterogeneity. J. Phys. Chem. B.

[42] Lilley D.M.J., Norman D.G. (1999). The Holliday Junction Is Finally Seen with Crystal Clarity. Nat. Struct. Biol..

[43] Simmons C.R.R., MacCulloch T., Krepl M., Matthies M., Buchberger A., Crawford I., Šponer J., Šulc P., Stephanopoulos N., Yan H. (2022). The Influence of Holliday Junction Sequence and Dynamics on DNA Crystal Self-Assembly. Nat. Commun..

[44] Stadler A.L., Renikuntla B.R., Yaron D., Fang A.S., Armitage B.A. (2011). Substituent Effects on the Assembly of Helical Cyanine Dye Aggregates in the Minor Groove of a DNA Template. Langmuir.

[45] Mass O.A., Wilson C.K., Roy S.K., Barclay M.S., Patten L.K., Terpetschnig E.A., Lee J., Pensack R.D., Yurke B., Knowlton W.B. (2020). Exciton Delocalization in Indolenine Squaraine Aggregates Templated by DNA Holliday Junction Scaffolds. J. Phys. Chem. B.

[46] Kolosova O.S., Shishkina S.V., Marks V., Gellerman G., Hovor I.V., Tatarets A.L., Terpetschnig E.A., Patsenker L.D. (2019). Molecular Structure and Spectral Properties of Indolenine Based Norsquaraines versus Squaraines. Dye. Pigment..

[47] Kuster S., Geiger T. (2015). Coupled π-Conjugated Chromophores: Squaraine Dye Dimers as Two Connected Pendulums. Dye. Pigment..

[48] Ilina K., MacCuaig W.M., Laramie M., Jeouty J.N., Mcnally L.R., Henary M. (2020). Squaraine Dyes: Molecular Design for Different Applications and Remaining Challenges. Bioconjug. Chem..

[49] Biaggne A., Knowlton W.B., Yurke B., Lee J., Li L. (2021). Substituent Effects on the Solubility and Electronic Properties of the Cyanine Dye Cy5: Density Functional and Time-Dependent Density Functional Theory Calculations. Molecules.

[50] Barcenas G., Biaggne A., Mass O.A., Wilson C.K., Obukhova O.M., Kolosova O.S., Tatarets A.L., Terpetschnig E., Pensack R.D., Lee J. (2021). First-Principles Studies of Substituent Effects on Squaraine Dyes. RSC Adv..

[51] Barclay M.S., Roy S.K., Huff J.S., Mass O.A., Turner D.B., Wilson C.K., Kellis D.L., Terpetschnig E.A., Lee J., Davis P.H. (2021). Rotaxane Rings Promote Oblique Packing and Extended Lifetimes in DNA-Templated Molecular Dye Aggregates. Commun. Chem..

[52] Barclay M.S., Wilson C.K., Roy S.K., Mass O.A., Obukhova O.M., Svoiakov R.P., Tatarets A.L., Chowdhury A.U., Huff J.S., Turner D.B. (2022). Oblique Packing and Tunable Excitonic Coupling in DNA-Templated Squaraine Rotaxane Dimer Aggregates. ChemPhotoChem.

[53] Jang Y.H., Hwang S., Kim Y.H., Jang S.S., Goddard W.A. (2004). Density Functional Theory Studies of the [2]Rotaxane Component of the Stoddart-Heath Molecular Switch. J. Am. Chem. Soc..

[54] Fothergill J.W., Hernandez A.C., Knowlton W.B., Yurke B., Li L. (2018). Ab Initio Studies of Exciton Interactions of Cy5 Dyes. J. Phys. Chem. A.

[55] Stennett E.M.S., Ma N., van der Vaart A., Levitus M. (2014). Photophysical and Dynamical Properties of Doubly Linked Cy3–DNA Constructs. J. Phys. Chem. B.

[56] Cunningham P.D., Kim Y.C., Sebastián S., Díaz S.A., Buckhout-White S., Mathur D., Medintz I.L., Melinger J.S. (2018). Optical Properties of Vibronically Coupled Cy3 Dimers on DNA Scaffolds. J. Phys. Chem. B.

[57] Biaggne A., Kim Y.C., Melinger J.S., Knowlton W.B., Yurke B., Li L. (2022). Molecular Dynamics Simulations of Cyanine Dimers Attached to DNA Holliday Junctions. RSC Adv..

[58] Biaggne A., Spear L., Ketteridge M., Kim Y.C., Melinger J.S., Knowlton W.B., Yurke B., Li L. (2022). Data-Driven and Multiscale Modeling of DNA-Templated Dye Aggregates. Molecules.

[59] Iyer M., Li Z., Jaroszewski L., Sedova M., Godzik A. (2020). Difference Contact Maps: From What to Why in the Analysis of the Conformational Flexibility of Proteins. PLoS ONE.

[60] Bhattacharya S., Bhattacharya D. (2020). Evaluating the Significance of Contact Maps in Low-Homology Protein Modeling Using Contact-Assisted Threading. Sci. Rep..

[61] Best R.B., Hummer G., Eaton W.A. (2013). Native Contacts Determine Protein Folding Mechanisms in Atomistic Simulations. Proc. Natl. Acad. Sci. USA.

[62] Basu S., Cervantes-Salguero K., Yurke B., Knowlton W.B., Lee J., Mass O.A. (2022). Photocrosslinking Probes Proximity of Thymine Modifiers Tethering Excitonically Coupled Dye Aggregates to DNA Holliday Junction. Molecules.

[63] Uy J.L., Asbury C.L., Petersen T.W., Van Den Engh G. (2004). The Polarization of Fluorescence of DNA Stains Depends on the Incorporation Density of the Dye Molecules. Cytom. Part A.

[64] Duarte D.J.R., Sosa G.L., Peruchena N.M., Alkorta I. (2016). Halogen Bonding. The Role of the Polarizability of the Electron-Pair Donor. Phys. Chem. Chem. Phys..

[65] Bresme F., Wynveen A. (2007). On the Influence of Solute Polarizability on the Hydrophobic Interaction. J. Chem. Phys..

[66] Sugita Y., Okamoto Y. (1999). Replica-Exchange Molecular Dynamics Method for Protein Folding. Chem. Phys. Lett..

[67] Husic B.E., Pande V.S. (2018). Markov State Models: From an Art to a Science. J. Am. Chem. Soc..

[68] Bauer P., Hess B., Lindahl E. (2022). GROMACS 2022.1 Manual. Zenodo.

[69] Bekker H., Benendsen H.J.C., Dijkstra E.J., Achterop S., Vondrumen R., Vanderspoel D., Sijbers A., Keegstra H., Renardus M.R., Nadrchal R.D., Nadrchal J. (1993). Gromacs—A Parallel Computer for Molecular-Dynamics Simulations.

[70] Graen T., Hoefling M., Grubmü H. (2014). AMBER-DYES: Characterization of Charge Fluctuations and Force Field Parameterization of Fluorescent Dyes for Molecular Dynamics Simulations. J. Chem. Theory Comput..

[71] Wang J., Wolf R.M., Caldwell J.W., Kollman P.A., Case D.A. (2004). Development and Testing of a General Amber Force Field. J. Comput. Chem..

[72] Bayly C.I., Cieplak P., Cornell W.D., Kollman P.A. (1993). A Well-Behaved Electrostatic Potential Based Method Using Charge Restraints for Deriving Atomic Charges: The RESP Model. J. Phys. Chem..

[73] Leland B., Paul D., Krueger B., Walker R. Tutorial A1: Setting up an Advanced System (Including Basic Charge Derivation). https://ambermd.org/tutorials/advanced/tutorial1/index.php.

[74] Sims M., Smith B., Helfrich B., Sathaye N., Messick E., Moore T., Fish R., Rajagopalan M., Rotkiewicz P., Drexler K.E. Nanoengineer-1—A CAD-Based Molecular Modeling Program for Structural DNA Nanotechnology. Proceedings of the Foundations of Nanoscience: Self-Assembled Architectures and Devices 2008 (FNANO08).

[75] Rappé A.K., Casewit C.J., Colwell K.S., Goddard W.A., Skiff W.M. (1992). UFF, a Full Periodic Table Force Field for Molecular Mechanics and Molecular Dynamics Simulations. J. Am. Chem. Soc..

[76] Wheatley E.G., Pieniazek S.N., Mukerji I., Beveridge D.L. (2012). Molecular Dynamics of a DNA Holliday Junction: The Inverted Repeat Sequence d(CCGGTACCGG) 4. Biophys. J..

[77] Yu J. (2004). Conformational Model of the Holliday Junction Transition Deduced from Molecular Dynamics Simulations. Nucleic Acids Res..

[78] Watson J., Hays F.A., Ho P.S. (2004). Definitions and Analysis of DNA Holliday Junction Geometry. Nucleic Acids Res..

[79] Maier J.A., Martinez C., Kasavajhala K., Wickstrom L., Hauser K.E., Simmerling C. (2015). Ff14SB: Improving the Accuracy of Protein Side Chain and Backbone Parameters from Ff99SB. J. Chem. Theory Comput..

[80] Hess B., Bekker H., Berendsen H.J.C.C., Fraaije J.G.E.M.E.M. (1997). LINCS: A Linear Constraint Solver for Molecular Simulations. J. Comput. Chem..

[81] Goga N., Rzepiela A.J., De Vries A.H., Marrink S.J., Berendsen H.J.C. (2012). Efficient Algorithms for Langevin and DPD Dynamics. J. Chem. Theory Comput..

[82] Beyerle E.R., Dinpajooh M., Ji H., von Hippel P.H., Marcus A.H., Guenza M.G. (2021). Dinucleotides as Simple Models of the Base Stacking-Unstacking Component of DNA ‘Breathing’ Mechanisms. Nucleic Acids Res..

[83] Michaud-Agrawal N., Denning E.J., Woolf T.B., Beckstein O. (2011). MDAnalysis: A Toolkit for the Analysis of Molecular Dynamics Simulations. J. Comput. Chem..

[84] Gowers R., Linke M., Barnoud J., Reddy T., Melo M., Seyler S., Domański J., Dotson D., Buchoux S., Kenney I. MDAnalysis: A Python Package for the Rapid Analysis of Molecular Dynamics Simulations. Proceedings of the 15th Python in Science Conference.

[85] Daura X., Gademann K., Jaun B., Seebach D., van Gunsteren W.F., Mark A.E., Rigault A., Siegel J., Harrowfield J., Chevrier B. (1998). Peptide Folding: When Simulation Meets Experiment.

