# Supplementary material for: Molecular Dynamic Studies of Dye–Dye and Dye–DNA Interactions Governing Excitonic Coupling in Squaraine Aggregates Templated by DNA Holliday Junctions
Source: Int J Mol Sci. 2023 Feb 17;24(4):4059. doi: 10.3390/ijms24044059 (PMC9967300; doi:10.3390/ijms24044059)
Supplement: Supplementary file 1 [file ijms-24-04059-s001.zip › ijms-2182513-supplementary.pdf]

Molecular Dynamic Studies of Dye-Dye and Dye-DNA Interactions Governing Excitonic Coupling in Squaraine Aggregates Templated by DNA Holliday Junctions

Supplemental Information

German Barcenas[a], Austin Biaggne[a], Olya A. Mass[a], William B. Knowlton[ab], Bernard Yurke[ab], Lan Li*[ac]

[a] Micron School of Materials Science and Engineering, Boise State University, Boise, ID 83725, USA

[b] Department of Electrical and Computer Engineering, Boise State University, Boise, ID 83725, USA

[c] Center for Advanced Energy Studies, Idaho Falls, ID 83401, USA

* Corresponding Author: lanli@boisestate.edu

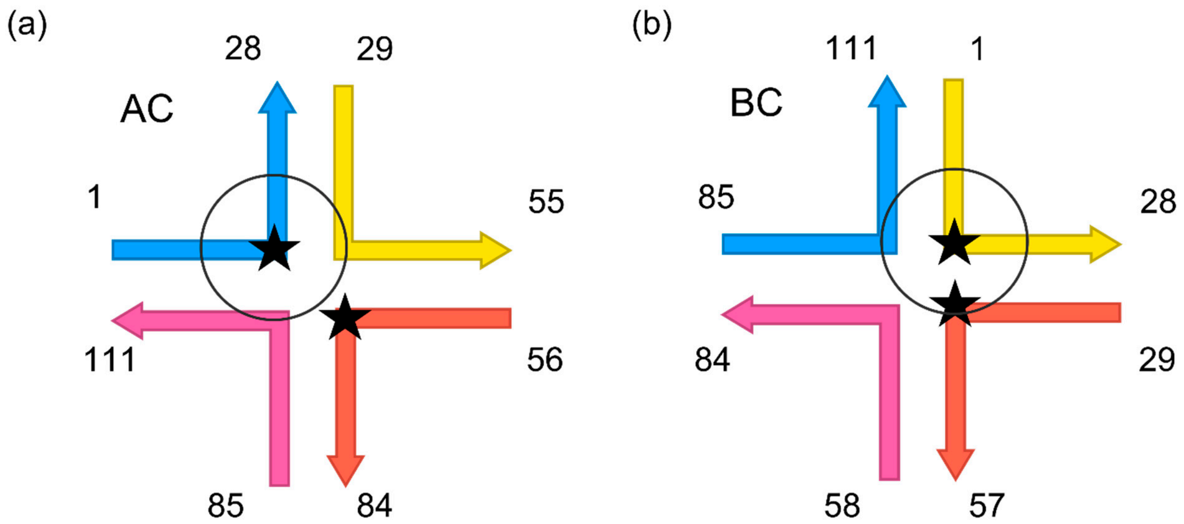

Figure S1: (a) AC and (b) BC HJ with each starting and ending residue ID for each strand, where the top left blue arrow is strand A, the top right yellow arrow is strand B, the bottom right orange arrow is strand C, and bottom left pink arrow is strand D. All arrows are pointing from the 5' to 3' direction. The circle found around residue 15 represents the contact circle with a radius of 1.2 nm we chose for the contact maps shown in Figures 3, S2 and S3.

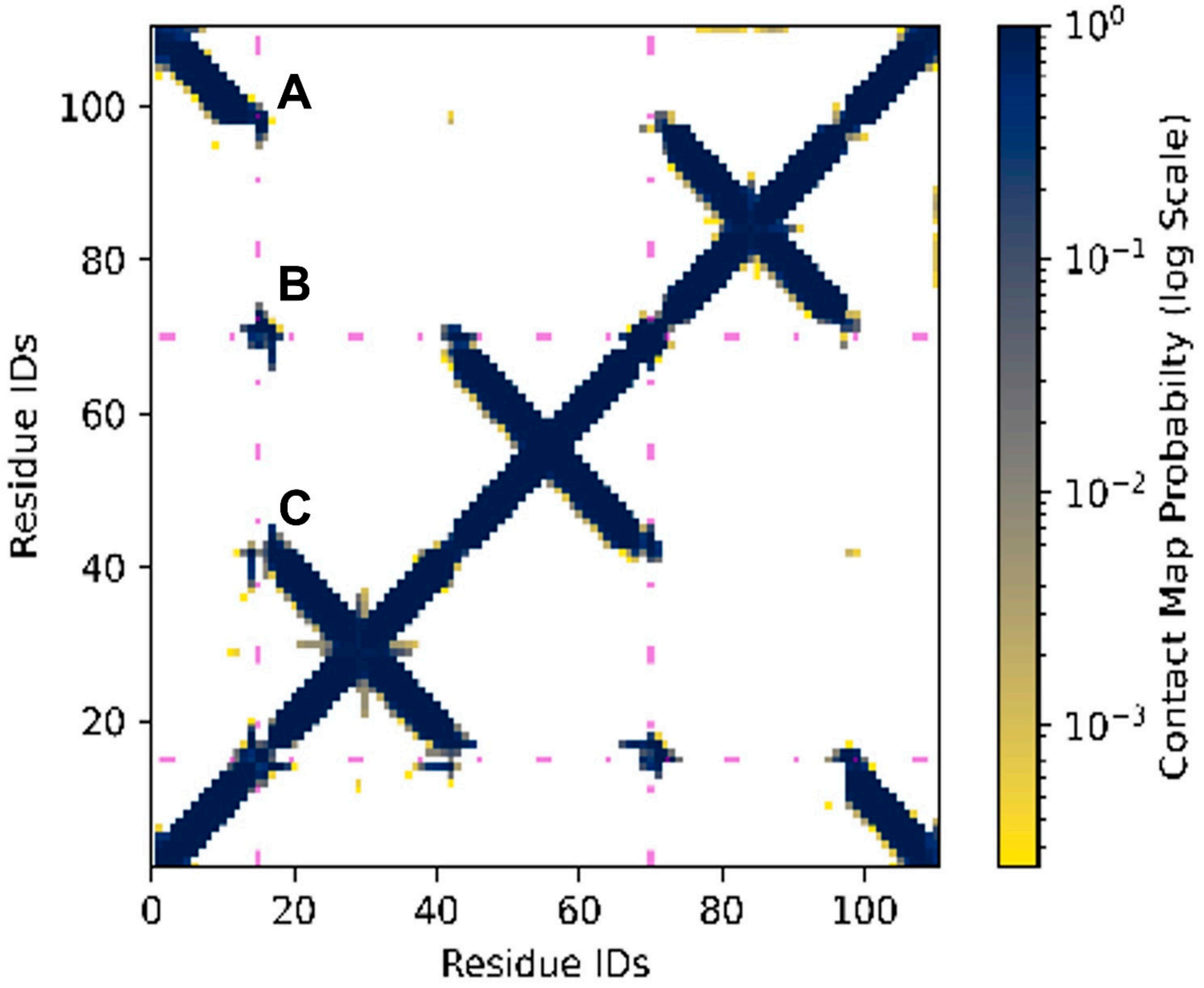

Figure S2: Contact Map for SQ-H_2 AC dimer and AB stacking - a bigger image for Figure 3a - with labels A, B and C representing the regions where a dye contacts. Residues IDs 15 and 70 represent the positions of two dyes. The dye residues are marked by magenta dashes. Contact map probability is represented by the log color scale, where darker colors represent more contact time, and lighter colors represent less contact. The regions of interest are labeled alphabetically to improve the plot's ease of reading.

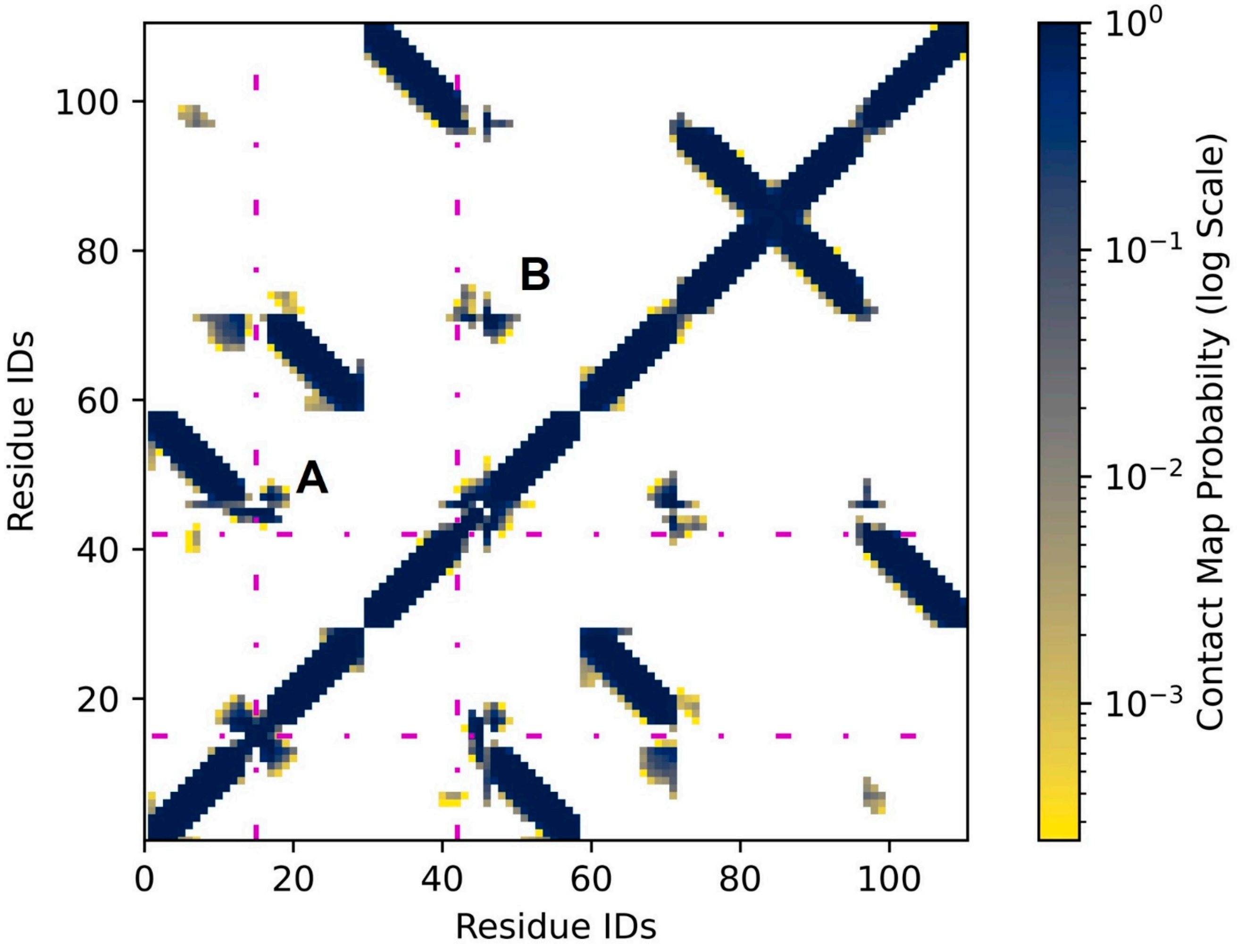

Figure S3: Contact Map for SQ-H_2 BC dimer and AB stacking - a bigger image for Figure 3b - with labels A and B representing the regions where two dye contact, respectively. For the BC dimers, residue IDs 15 and 42 represent the dyes. The dye residues are marked by magenta dashes. Contact map probability is represented by the log color scale, where darker colors represent more contact time, and lighter colors represent less contact. The regions of interest are labeled alphabetically to improve the plot's ease of reading.

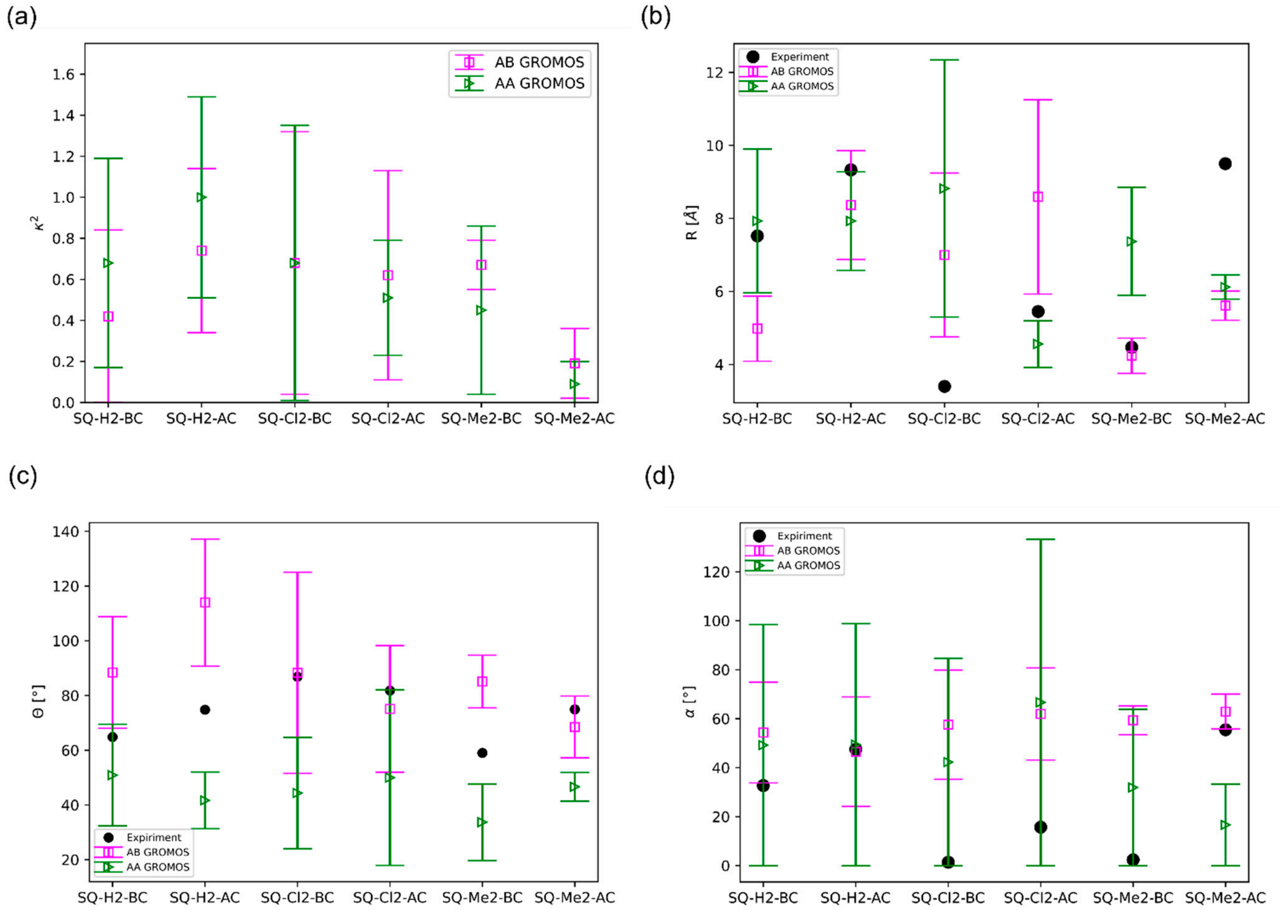

Figure S4: Orientation values for each dimer and stacking motif from GROMOS values, including (a) Orientation factor κ^2, (b) Dye-dye distance R, (c) Slip angle θ, and (d) Tilt angle α. The experimental values are adapted from Mass et al. [14]

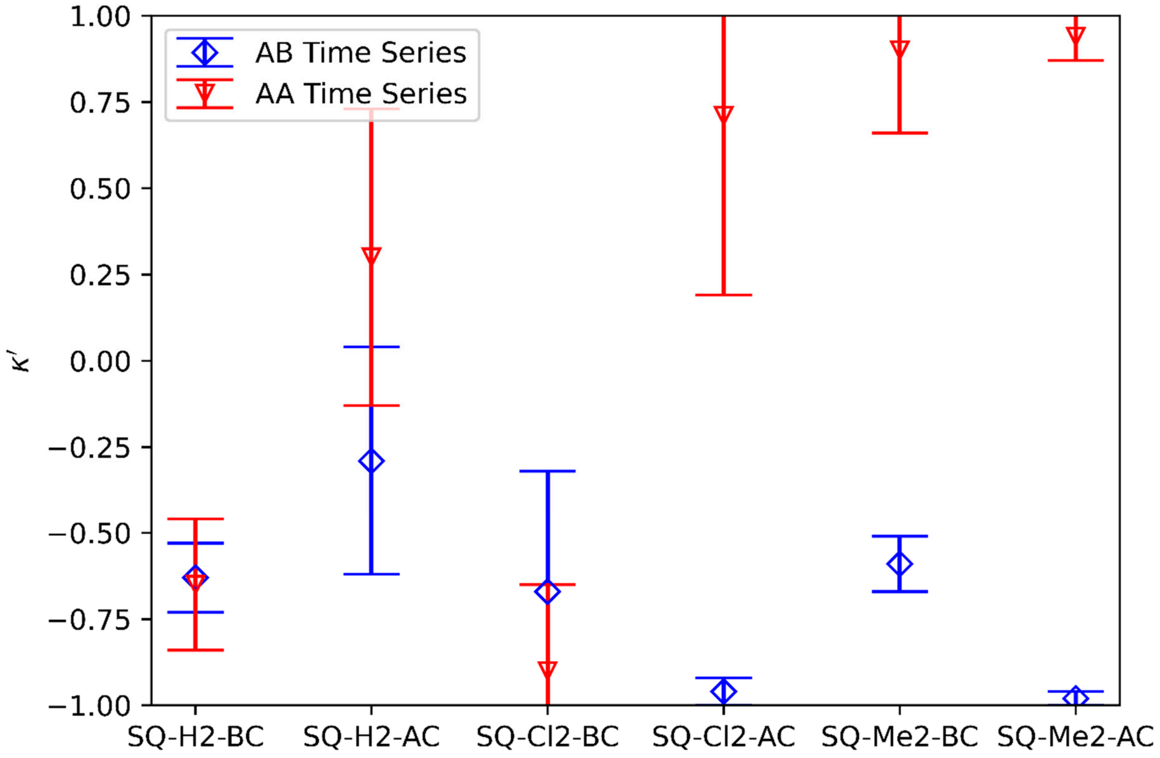

Figure S5: κ' for all SQ aggregates Time Series average data with standard deviation bars for each simulation.

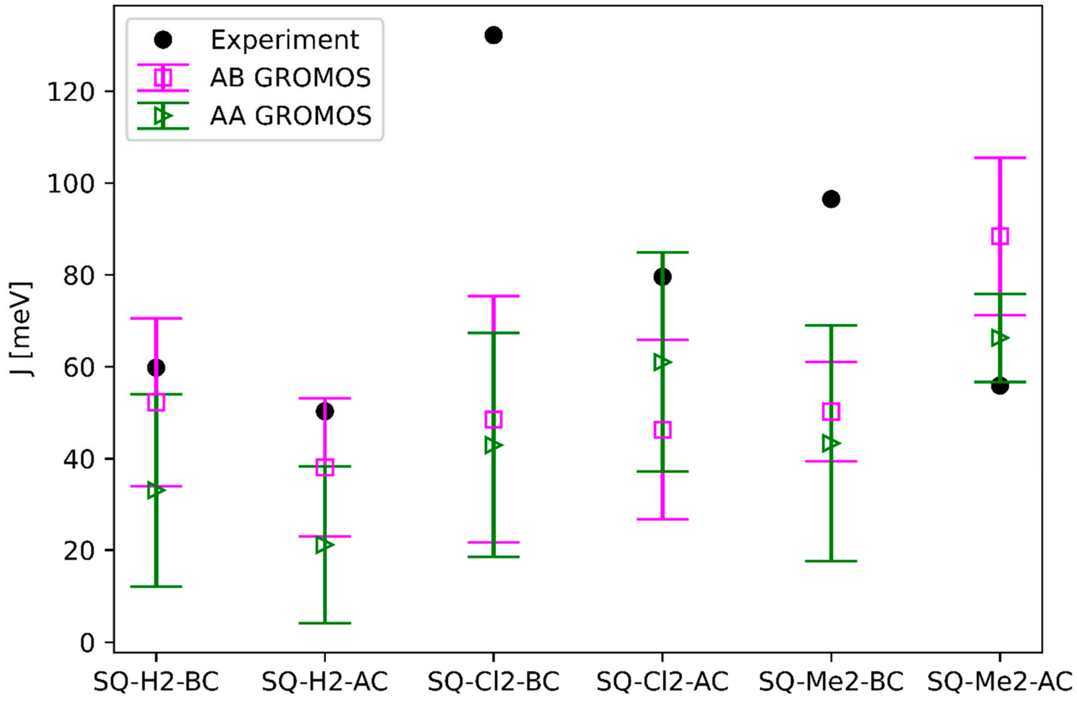

Figure S6: $J_{m,n}$ for all SQ aggregates GROMOS averages with standard deviation bars for each simulation. The experimental values are adapted from Mass et al. [14].
